# Ablation of the GDP-fucose transporter suppresses lung cancer cell proliferation and migration by reducing expression of PD-L1

**DOI:** 10.7150/jca.84652

**Published:** 2023-10-02

**Authors:** Yingshu Zhang, Nianzhu Zhang, Wanli Song, Sabiha Yousuf, Wenzhe Li

**Affiliations:** 1 Department of Thoracic Surgery, Cancer Hospital of Shantou University Medical College, Shantou, Guangdong, 515041, China.; 2 Affiliated Zhongshan Hospital of Dalian University, Dalian, Liaoning, 116001, China.; 3 Second Affiliated Hospital of Dalian Medical University, Dalian, Liaoning, 116044, China.; 4 College of Basic Medical Sciences, Dalian Medical University, Dalian, Liaoning 116044, China.

**Keywords:** Fucosylation, GDP-fucose transporter, Lung adenocarcinoma, PD-L1 ubiquitination, EGFR

## Abstract

Fucosylation, an important post-translational modification, is closely related to the development of tumors. In the microenvironment of lung cancer, expression of PD-L1 and fucosylation is abnormally upregulated. However, the correlation between PD-L1 expression and its fucosylation in lung adenocarcinoma (LUAD) remains unclear. The GDP-fucose transporter (GFT) is a key molecule in cellular fucosylation. To explore the correlation between fucosylation and PD-L1 expression, we knocked out the GFT-encoding gene *SLC35C1* in mouse Lewis lung adenocarcinoma cells and in human H1299 lung adenocarcinoma cells. Loss of *SLC35C1* impaired the phosphorylation of EGFR and its downstream target ERK. Moreover, loss of *SLC35C1* up-regulated the expression of β-TrCP, a PD-L1 E3 ligase, thereby promoting the ubiquitination of PD-L1 and its subsequent degradation. The down-regulated expression of PD-L1 leads to a decline in lung cancer cell proliferation and migration. These results suggest that fucosylation partially influences LUAD tumorigenesis by regulating PD-L1 expression.

## Introduction

Lung cancer is a disease characterized by high morbidity and mortality 1. Lung adenocarcinoma (LUAD) is the main pathologic type of lung cancer 2. As the crucial immune checkpoint, the programmed cell death-1 (PD-1)/PD-1 ligand 1 (PD-L1) axis contributes to lung cancer immunity. In view of the important role of PD-1/PD-L1, many anti-PD-1 or anti-PD-L1 antibodies have been produced for treating lung cancer via blockading the PD-1/PD-L1 pathway 3.

**Figure 1:**
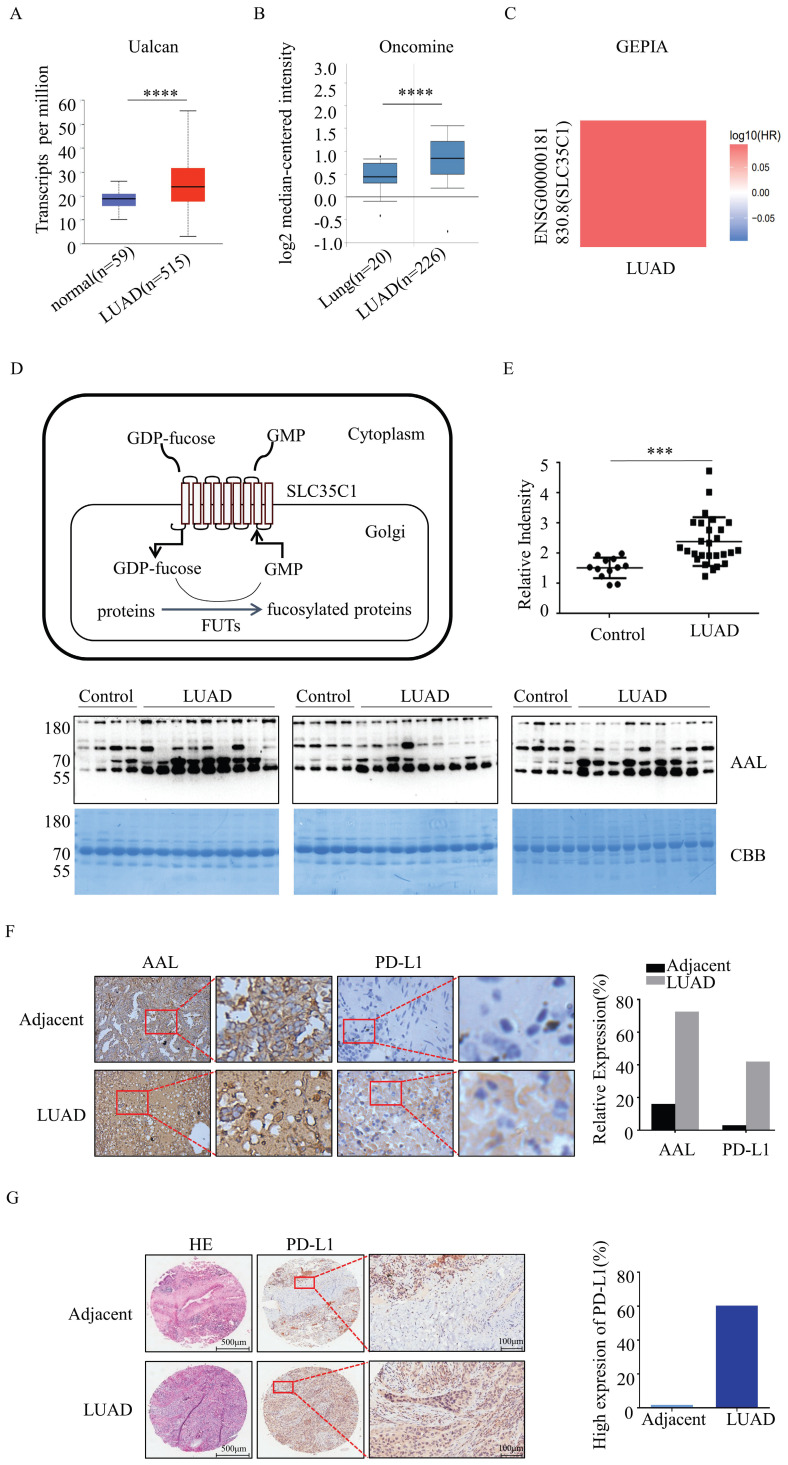
Fucosylation and PD-L1 expression are upregulated in LUAD. (A) Expression of SLC35C1 mRNA in LUAD compared to normal individuals, derived from the UALCAN database. **** p<0.0001. (B) SLC35C1 mRNA expression in LUAD compared to normal individuals, derived from the Oncomine database. **** p<0.0001. (C) Correlation between SLC35C1 and OS (overall survival) of LUAD patients was evaluated by using the GEPIA database. (D) Protein fucosylation pathway. GFT is a 10-transmembrane GDP-fucose transporter and couples the import of GDP-fucose into the Golgi lumen. In the Golgi apparatus, FUTs catalyze the transfer of the fucose residue from a GDP-fucose donor to a protein. (E) Comparative analysis of the fucosylation levels in sera. Serum samples were run on a 10% SDS-PAGE gel. Fucosylation levels in sera of 27 LUAD patients and 12 healthy donors were detected by AAL blotting. The gel was stained with Coomassie brilliant blue (CBB). *** p<0.001. Data are shown as mean ± SD and are representative of three independent experiments. (F) Immunohistochemical analysis of PD-L1 and fucosylation. Slides were incubated with anti-PD-L1 Ab (1:150) or AAL. The magnifications are 10X and 40X, respectively. The brown color indicates positive staining for PD-L1 expression and fucosylation. (G) Immunohistochemical analysis of PD-L1 expression in a LUAD tissue microarray (n=92). Representative images of PD-L1 level in a LUAD and adjacent tissue are shown. Microarrays were incubated with anti-PD-L1 Ab (1:150). After washing, secondary antibody was incubated and visualized with DAB. Data are shown as mean ± SD of triplicates and are representative of three independent experiments.

Fucosylation is one of the most common post-translational modifications. In mammals, fucose is incorporated into N-glycans, O-glycans and glycolipids by 13 different fucosyltransferases (FUTs). Ten fucosyltransferases (FUT1-7 and FUT9-11) are responsible for the addition of a terminal fucose to glycan chains, whereas FUT8 is the sole enzyme responsible for catalyzing core fucosylation. The other two protein O-fucosyltransferases, POFUT1 (FUT12) or POFUT2 (FUT13) are localized in the ER 4. As the fucose donor used by fucosyltransferases, guanosine 5-diphosphate (GDP)-fucose is synthesized in the cytosol via both the salvage pathway and the de novo pathway. The de novo pathway is the major pathway and utilizes two key enzymes involving GDP-mannose 4,6-dehydratase (GMD) and GDP-4-keto-6-deoxy-mannose-3,5-epimerase-4-reductase (FX). Then, GDP-fucose is transferred from the cytosol into the Golgi apparatus by the GDP-fucose transporter (GFT) encoded by the *SLC35C1* gene (Fig. 1D), with lysine^303^ and threonine^308^, located near the C-terminus of GFT, participating in the recognition of the GDP-fucose 5. A hereditary deficiency of SLC35C1 leads to a generalized loss of fucosylated glycans on the cell surface and results in leukocyte adhesion deficiency (LAD) type II syndrome 6, 7. Aberrant fucosylation is associated with a variety of tumors, including lung, liver and colorectal cancers 8, 9. Dysregulation of core fucosylation would be a potential biomarker or prognostic indicator for lung cancer 10, 11.

The PD-1/PD-L1 pathway plays a crucial role in tumorigenesis 12, 13. PD-L1 is a glycoprotein expressed on tumor cells, and has four N-glycosylation sites, N^35^, N^192^, N^200^, and N^219^, that regulate PD-L1 stability 14. So far, studies on the regulation of PD-L1 expression have focused on transcriptional control by MYC, ALK, NF-κB, and the IFN-γ/JAK/STAT1 pathway. Moreover, phosphorylation of PD-L1 by GSK3β is associated with degradation of PD-L1 14,15. However, few studies have focused on the regulation of PD-L1 by post-translational modifications, such as glycosylation and ubiquitination. In the present study, we first found that both fucosylated proteins and PD-L1 are highly expressed in LUAD tissues. In order to investigate the correlation between fucosylation and PD-L1 expression in LUAD tumorigenesis, we knocked out the SLC35C1 gene by using the CRISPR/Cas9 system. Our data suggests that loss of SLC35C1 suppresses lung cancer cell migration and proliferation by reducing PD-L1 expression.

## Materials and Methods

### Antibodies (Abs)

Anti-mouse PD-L1 Ab (EPR20529) (ab213480) was purchased from Abcam (Cambridge, UK). Anti-human PD-L1 Ab (E1L3N), anti-β-TrCP mAb (D13F10), anti-phospho-Erk1/2 (Thr202/Tyr204) Ab (9101), p44/42 MAPK (Erk1/2) Ab (9102), cyclin E1 (HE12) mouse mAb (4129) and cyclin B1 (V152) mouse mAb (4135) were from Cell Signaling Technology (Beverly, MA, USA). Rabbit polyclonal anti-SPOP Ab and anti-glyceraldehyde-3-phosphate dehydrogenase (GAPDH) Ab (1E6D9) were purchased from the Proteintech Group (Chicago, IL, USA). Anti-ubiquitin Ab (P4D1) was purchased from Santa Cruz Biotechnology. Mouse anti-EGF receptor (610017) was purchased from BD Transduction Laboratories. Anti-SLC35C1 Ab (PA564146) was purchased from Thermo Fisher Scientific. Horseradish peroxidase (HRP)-labeled goat anti-mouse IgG and HRP-conjugated goat anti-rabbit IgG were purchased from Beyotime (Shanghai, China).

### Clinical samples

Serum specimens were collected from 27 patients with LUAD and 12 healthy donors. The diagnosis of lung adenocarcinoma was made based on clinical manifestation, serology, imaging and histopathology. Serum specimens were from patients between April 2017 and April 2019 in Tianjin Medical University Cancer Institute & Hospital, Tianjin, China. The Ethics Committee at the hospital approved the study (No. Bc2019025). Human lung adenocarcinoma tissue microarrays, including 92 lung adenocarcinomas (41 females and 51 males) and their corresponding adjacent tissues, were analyzed by the Outdo Biotech Company (Shanghai, China). Immunohistochemistry was carried out to analyze the level of PD-L1 expression. All patients had complete surgical resection and were recruited between July 2008 and June 2013 in China. Detailed clinical parameters of the enrolled patients are listed in Table S1. The postoperative pathological diagnosis was LUAD. The exclusion criteria were as follows: (1) had other serious diseases; (2) received other treatments, including preoperative chemotherapy, neoadjuvant chemotherapy, or immunotherapy. Informed consent for all subjects were exempted by an ethics committee (Acceptance No. YBM-05-01).

### Cell culture

Lewis lung carcinoma, H1299 and 293T cells were obtained from the American Type Culture Collection (ATCC). Lewis lung carcinoma cells were maintained in Dulbecco's modified Eagle's medium (Gibco, USA) supplemented with 10% fetal bovine serum (FBS) (Gibco), penicillin and streptomycin (Sangon; Shanghai, China). H1299 and 293T cells were maintained in RPMI 1640 medium (Gibco) supplemented with 10% FBS, penicillin and streptomycin. All cells were maintained in a humidified incubator with 5% CO_2_ at 37°C.

### Immunohistochemistry

Relative protein expressions in LUAD samples were analyzed by immunohistochemistry. Briefly, tissue slides were deparaffinized in xylene and hydrated through a 100%, 90%, 80% and 70% ethanol series to PBS, and were incubated with 3% H_2_O_2_ for 30 min and blocked using an avidin/biotin blocking kit. Anti-human PD-L1 Ab (1:200) or biotinylated Aleuria aurantia lectin (AAL) (Vector Laboratories) were incubated for 1 hr. Then, sections were incubated with secondary antibody for 1 hour (hr), and then treated with 3,3'-diaminobenzidine (DAB) (Solarbio, China). Expression of AAL and PD-L1 was assessed by determining staining intensity, which was analyzed by integrated optical density using Image-Pro Plus software (version 6.0; Media Cybernetics, USA).

### Establishment of SLC35C1 gene knockout Lewis lung adenocarcinoma cells

SLC35C1 gene knockout cells were generated using the CRISPR/Cas9 system. Two guide RNAs (gRNAs) for the mouse *SLC35C1* gene (NC-000068.8), gRNA1: 5'-CGGGCTCTGCAGATCGCGC-3' (1427-1445 bp) and gRNA2: 5'-CGGTCCAGGATCCTGCGCA-3' (1342-1360 bp) were designed by GenScript. The pGS-U6-gRNA (zeocin resistance) and pGS-CMV-hcas9 (neomycin resistance) plasmids were co-transfected into Lewis lung adenocarcinoma cells with Lipofectamine 2000 (Invitrogen). SLC35C1 gene knockout Lewis (Lewis-KO) cells were generated after selection with 400 μg/ml G418 and 400 μg/ml zeomycin. To verify the success of SLC35C1 gene knock out, the genomic DNA of the Lewis-KO cells was extracted, and the DNA sequence was then analyzed by Sangon (Shanghai, China), using SLC35C1 gene primers 5'-CCAAGAAGGAGGGGACCGAT-3' (sense); 5'-AGAAGGTGACAAAAATGG GGGT-3' (antisense).

### Establishment of H1299-KD and H1299-KD-Re cells

The pLKO.1 shRNA lentivirus system (CS-SH3263-LVRU6GP) was used to generate SLC35C1 gene knockdown in H1299 cells. The shRNA for the SLC35C1 gene (sense: 5'-AGACCTTGATACTTTCAAA-3', antisense: 5'-TTTGAAAGTATCAAGGTCT-3') was designed by GeneCopoeia (Guangzhou, China). The lentivirus vectors were transfected into 293 T cells and the cells were cultured for 48 hours (hrs). SLC35C1 gene knockdown H1299 (H1299-KD) cells, following transduction by lentivirus expressing SLC35C1 shRNA, were selected in puromycin (3 μg/ml). For SLC35C1 reintroduction, full-length SLC35C1 cDNA was cloned into the pLHCX vector (Clontech). The pLHCX-SLC35C1 expression vector was transfected into H1299-KD cells to generate SLC35C1-restored (H1299-KD-Re) cells after selection with 400 μg/ml G418.

### Establishment of PD-L1 gene knockdown cells

The plasmid (CS-SH3334-LVRU6GP) encoding shRNA specific for PD-L1 was designed by GeneCopoeia (Guangzhou, China). For PD-L1 knock down, H1299 cells were seeded in 6-well plates overnight. Then the plasmid encoding shRNA (sense: 5'-AGAccuuGAuAcuuucAAAdTsdT-3', antisense: 5'-UUUGAAAGuAUcAAGGUCUdTsdT-3') was transfected into the cells using Lipofectamine 2000. Forty-eight hours after transfection, cells were selected with puromycin (3 μg/ml) and harvested for western blotting to quantify the PD-L1 expression.

### Cell stimulation

Cells were treated with 4 nM EGF or 20 nM PD98059 (HY-12028). Then, cells were washed twice with phosphate-buffered saline (PBS) and lysed with protein lysis buffer (50 mM Tris-HCl pH=8.0, 1% Trion X-100, 150 mM NaCl, 10% glycerol, 2 mM EDTA, 100 μM PMSF) for 30 min on ice. Lysates were centrifuged at 12,000 *g* for 15 min at 4°C and the supernatant was collected. Protein concentration was determined by bicinchoninic acid protein assay (BCA, Beyotime, China).

### Western blot and lectin blot analysis

Equal amounts of protein were separated by 10% sodium dodecyl sulfate-polyacrylamide gel electrophoresis (SDS-PAGE) and then transferred to polyvinylidene difluoride (PVDF) membranes (Millipore Corp). Membranes were blocked with 5% fat-free milk or BSA in TBS-T (10 mM Tris-HCl, 150 mM NaCl, and 0.1% Tween 20) for 1 hr at room temperature (RT), then incubated with appropriate primary Abs or biotinylated AAL for 1 hr at RT. After washing, membranes were incubated with the corresponding HRP-conjugated secondary Abs or HRP-conjugated streptavidin (Beyotime, Shanghai, China), and the proteins were visualized with an ECL system (Amersham, Sweden).

### Immunoprecipitation (IP)

Serum-starved cells were treated with 0, 1 and 4 nM of EGF. After washing with PBS contained 0.4 mM sodium orthovanadate, cells were solubilized for 15 min at 4°C in lysis buffer (50 mM Tris-HCl [pH=8.0], 1% [v/v] Triton X-100, 10% [v/v] glycerol, 2 mM EDTA, 100 μM phenylmethylsulfonylfluoride, 5 μg/ml of leupeptin, 1 μg/ml of aprotinin, 100 mM NaF, and 1 mM sodium orthovanadate). The lysate was centrifuged at 10,000 *g* for 10 min at 4°C to precipitate insoluble materials, and the supernatant was then subjected to immunoprecipitation, as indicated below.

For immunoprecipitation, the cell extracts (500 μg) were mixed with 20 μl of a 50% suspension of Protein G-Sepharose (Amersham Pharmacia Biotech AB) and incubated at 4°C with continuous rotation. Ten μl of Abs was added and incubated overnight at 4°C with gentle agitation. The beads were washed four times with lysis buffer, then immunoprecipitated samples were eluted from the protein G-Sepharose by heating at 100°C for 5 min in Laemmli sample buffer.

### Cell proliferation assay

Cell proliferation was assessed by MTT (3-(4,5-dimethylthiazol-2-yl)-2,5-diphenyltetrazolium bromide) assay. Cells were suspended in culture medium with 5% FBS and inoculated in a 96-well plate at 5 x 10^3^ cells/well. After incubation for 24, 48, 72 and 96 hrs, 10 µl of MTT solution was added to each well. Cells were incubated for an additional 4 hrs before removing the medium and adding 200 μl dimethylsulfoxide (DMSO) to each well and absorbance measured at 490 nm with a microplate reader (Thermo, Finland).

### Wound healing assay

Cells were inoculated in 6-well culture plates and allowed to reach 90% confluence. After serum starvation for 24 hrs, a sterile pipette tip was used to scratch the monolayer. Then, medium with 5% FBS was added to each plate. The same position was photographed at 0 and 24 hrs for later calculation by the microscope and DigitalCam Software. ImageJ software was used to measure and calculate the distance that the cells had migrated.

### Transwell migration assay

The migratory effect of SLC35C1 was evaluated in a 24-well, 8 μm pore size polycarbonate membrane transwell chamber (Corning, NY, USA) assay. A suspension of 2 × 10^5^ cells in 200 μl DMEM containing 5% FBS was inoculated into the upper transwell chamber, and the lower chamber contained 600 μl DMEM plus 10% FBS. The cells were incubated for 16 hrs. After removal with PBS of non-migrated cells on the upper layer of the membrane, cells that had migrated through the membrane were fixed in 4% paraformaldehyde and stained with 0.1% crystal violet.

### Quantitative real-time PCR

H1299 cells were lysed with RNAiso Plus reagent (TaKaRa, China) to extract total RNA, then cDNAs were synthesized using an Evo M-MLV RT kit (AG, China). Real-time PCR was performed using SYBR Green Master Mix and an Applied Biosystems Prism 7000 Sequence Detection System (Applied Biosystems, Japan). Primers for c-jun (sense: 5'-AAA CTG TGA GAC TGG GAA GTA G-3', antisense: 5'-CGA TCA CAA AGA TAA ACC GCA T-3') and c-fos (sense: 5'-TGC ACT GCT TAC ACG TCT TCC TTC-3', antisense: 5'-TCA TTG CTG CTG CTG CCC TTG-3') were used. GAPDH served as an internal control. The thermal cycling conditions for real-time PCR were 10 s at 95°C to activate SYBR Ex Taq, followed by 40 cycles of denaturation for 5 s at 95°C and annealing/extension for 30 s at 60°C. After the last cycle, a final extension was performed at 72°C for 5 min. Relative expression of the target genes to the internal control genes was calculated using the formula: relative expression = 2-^ΔCT^ (ΔCT = CT_targetgene_-CT _internalcontrol_).

### Statistical analysis

Data are presented as mean ± standard deviation (SD) from at least 3 independently repeated experiments. Two-tailed Student's *t*-tests were performed to compare the statistical differences between groups, using GraphPad Prism software version 7, and differences with p<0.05 were considered statistically significant. * p<0.05, ** p<0.01, *** p<0.001, **** p<0.0001.

## Results

### Fucosylation and PD-L1 expression are elevated in LUAD tissues

GFT encoded by the SLC35C1 gene is a key molecule that regulates the fucosylation. To understand the relationship between SLC35C1 and LUAD tumorigenesis, we analyzed the mRNA expression level of SLC35C1 in LUAD by using public databases. The mRNA expression of SLC35C1 was upregulated in LUAD tissues in the UALCAN database (p=1 × 10^-12^) (Fig. 1A) and Oncomine database (p<0.001) (Fig. 1B). Next, we used the GEPIA database to evaluate the prognostic value of SLC35C1 in survival of NSCLC patients (Fig. 1C). The GEPIA database is an online website, and can be used to analyze tumor and normal tissue data from TCGA. Higher SLC35C1 expression was associated with poorer overall survival in LUAD.

Moreover, fucosylated proteins were dramatically increased in the sera of LUAD patients (p<0.001), as evidenced by lectin blotting with AAL, which recognizes fucosylated structures 16 (Fig. 1E). In fresh clinical specimens, fucosylation was increased in LUAD tissues (Fig. 1F). Expression of PD-L1 was also increased in LUAD tissues (Fig. 1F), indicating that fucosylation correlates with PD-L1 expression. In the analysis of human lung adenocarcinoma tissue microarrays, there was elevated expression of PD-L1 in LUAD tissue compared with adjacent tissue (n=92) (Fig. 1G). Collectively, these data suggest that increased fucosylation and PD-L1 expression contributes to the tumorigenesis of LUAD.

### Loss of SLC35C1 gene down-regulates PD-L1 expression

**Figure 2:**
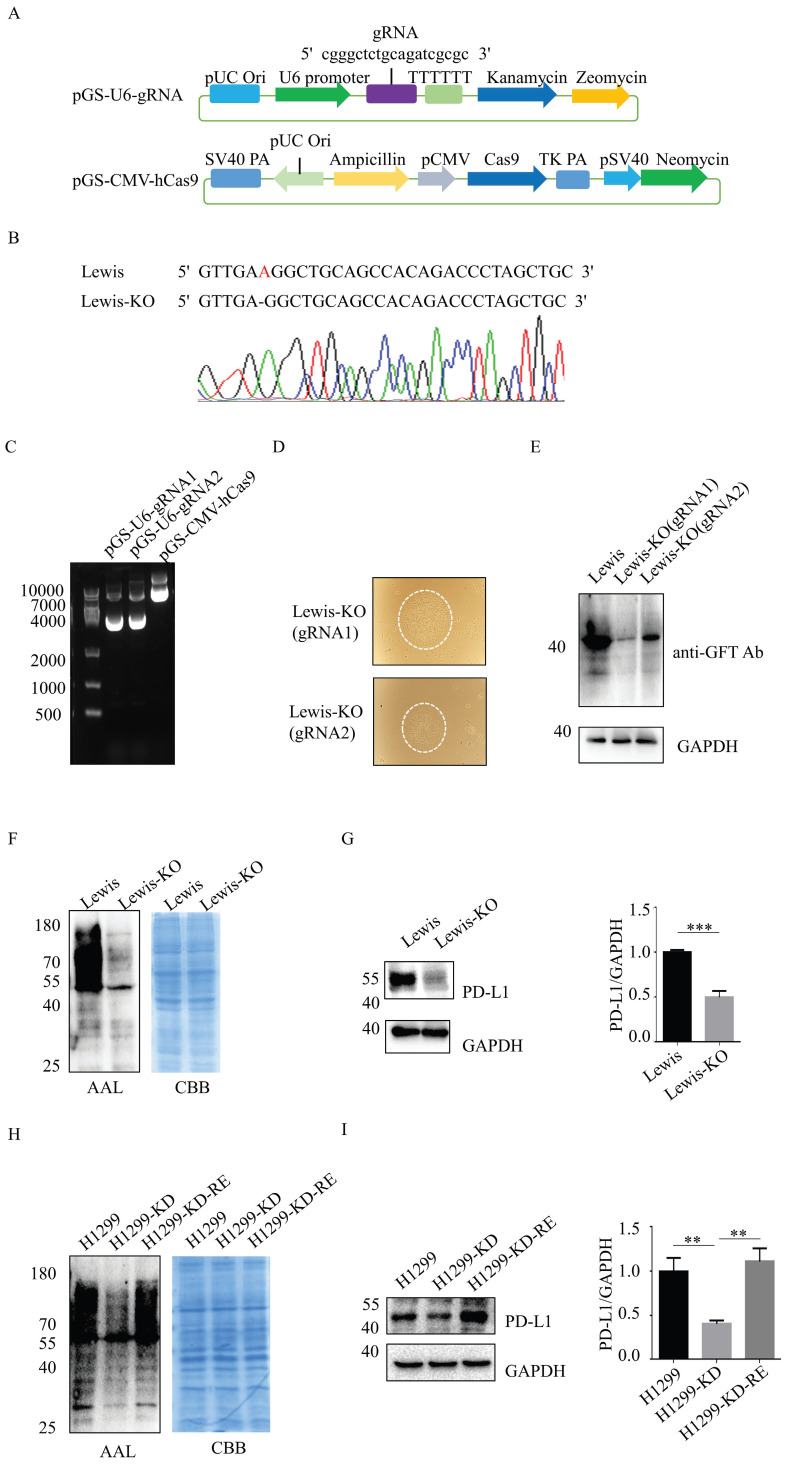
SLC35C1 knockdown suppresses PD-L1 expression. (A) The CRISPR/Cas9 system consisted of the pGS-CMV-hcas9 for Cas9 protein and pGS-U6-gRNA for the gRNA. We designed gRNA1 (5'-cgggctctgcagatcgcgc-3') for SLC35C1 gene knockout. (B) SLC35C1 gene sequence alignment. SLC35C1 knockout alleles were generated by CRISPR-Cas9 gene editing and resulted in deletion of an adenine base in Lewis-KO cells. (C) Two different gRNAs targeting the SLC35C1 gene were designed and named gRNA1 and gRNA2. The gRNA1, gRNA2 and Cas9 plasmids were run on an agarose gel. (D) CRISPR/Cas9 expression vectors were co-transfected into Lewis lung adenocarcinoma cells. Colonies were selected via G418 and zeomycin. Representative images of monoclonal cells transfected by gRNA1 and gRNA2 are shown. (E) Expression of the GDP-fucose transporter was characterized by western blotting. Cell lysates (10 μg) were run on a 10% SDS-PAGE gel and stained with anti-GFT Ab (1:1,000). (F) Lectin blot with AAL of Lewis-KO cells. Cell lysates (10 μg) were run on a 10% SDS-PAGE gel and stained with CBB and AAL (1:12,000). (G) PD-L1 expression in Lewis-KO cells. Cell lysates (10 μg) were separated on a 10% SDS-PAGE gel, transferred to a PVDF membrane and probed with anti-PD-L1-Ab (1:1,000). GAPDH was used as the loading control. (H) Establishment of H1299-KD and H1299-KD-Re cells. H1299 cells were transfected with pLKO.1 shRNA lentivirus encoding SLC35C1. H1299-KD cells were selected in puromycin. SLC35C1-restored H1299-KD-Re cells were generated by transfection of the pLHCX-SLC35C1 plasmid. Cell lysates (10 μg) were run on 10% SDS-PAGE gel and stained with CBB and AAL (1:12,000). (I) PD-L1 expression in H1299, H1299-KD and H1299-KD-Re cells. Cell lysates (10 μg) were run on 10% SDS-PAGE and stained with anti-PD-L1 Ab (1:1000). GAPDH was used as a loading control. Data are shown as mean ± SD and are representative of three independent experiments.

To assess whether fucosylation regulates PD-L1 expression in LUAD, we established SLC35C1 gene-knockout Lewis lung adenocarcinoma cells (Lewis-KO) using the CRISPR/Cas9 genome editing system. First, we designed specific SLC35C1-gRNA fragments complementary to different regions of the SLC35C1 open reading frame. The sequences of the gRNA corresponded to nucleotides 1427-1445 bp (gRNA1), and 1342-1360 bp (gRNA2) of the transfected cells (Fig. 2A). The pGS-U6-gRNA1 and pGS-CMV-hcas9 plasmids were then co-transfected into Lewis lung adenocarcinoma cells to silence SLC35C1 gene expression. A deletion of adenine base (1234 bp) was detected in Lewis-KO cells by DNA sequencing analysis (Fig. 2B). The pGS-U6-gRNA1, pGS-U6-gRNA2 and pGS-CMV-hcas9 plasmids were cloned and co-transfected into Lewis lung carcinoma cells (Fig. 2C), and clones from the Lewis-KO (gRNA1) cell line and Lewis-KO (gRNA2) cell line, were isolated (Fig. 2D). GDP-fucose transporter protein expression was decreased in Lewis-KO (gRNA1) cells compared to Lewis-KO (gRNA2) cells (Fig. 2E). Therefore, we chose Lewis-KO (gRNA1) cells for further study. As expected, fucosylation was barely detected in Lewis-KO cells (Fig. 2F). Simultaneously, PD-L1 expression was significantly reduced by knockout of SLC35C1 gene (Fig. 2G).

In addition, we knocked down SLC35C1 in human lung adenocarcinoma H1299 cells (H1299-KD). To restore the expression of SLC35C1, we designed the pLHCX-SLC35C1 vector and transfected it into H1299-KD cells. Reintroduction of the SLC35C1 gene into H1299-KD cells restored the fucosylation in H1299-KD-Re cells, and the levels were comparable to those in parental H1299 cells (Fig. 2H). PD-L1 expression was significantly reduced in H1299-KD cells, and was rescued by the reintroduction of SLC35C1 in H1299-KD-Re cells (Fig. 2I), indicating that PD-L1 expression is regulated by the SLC35C1 gene.

### Loss of SLC35C1 promotes PD-L1 ubiquitination by increased β-TrCP expression

**Figure 3:**
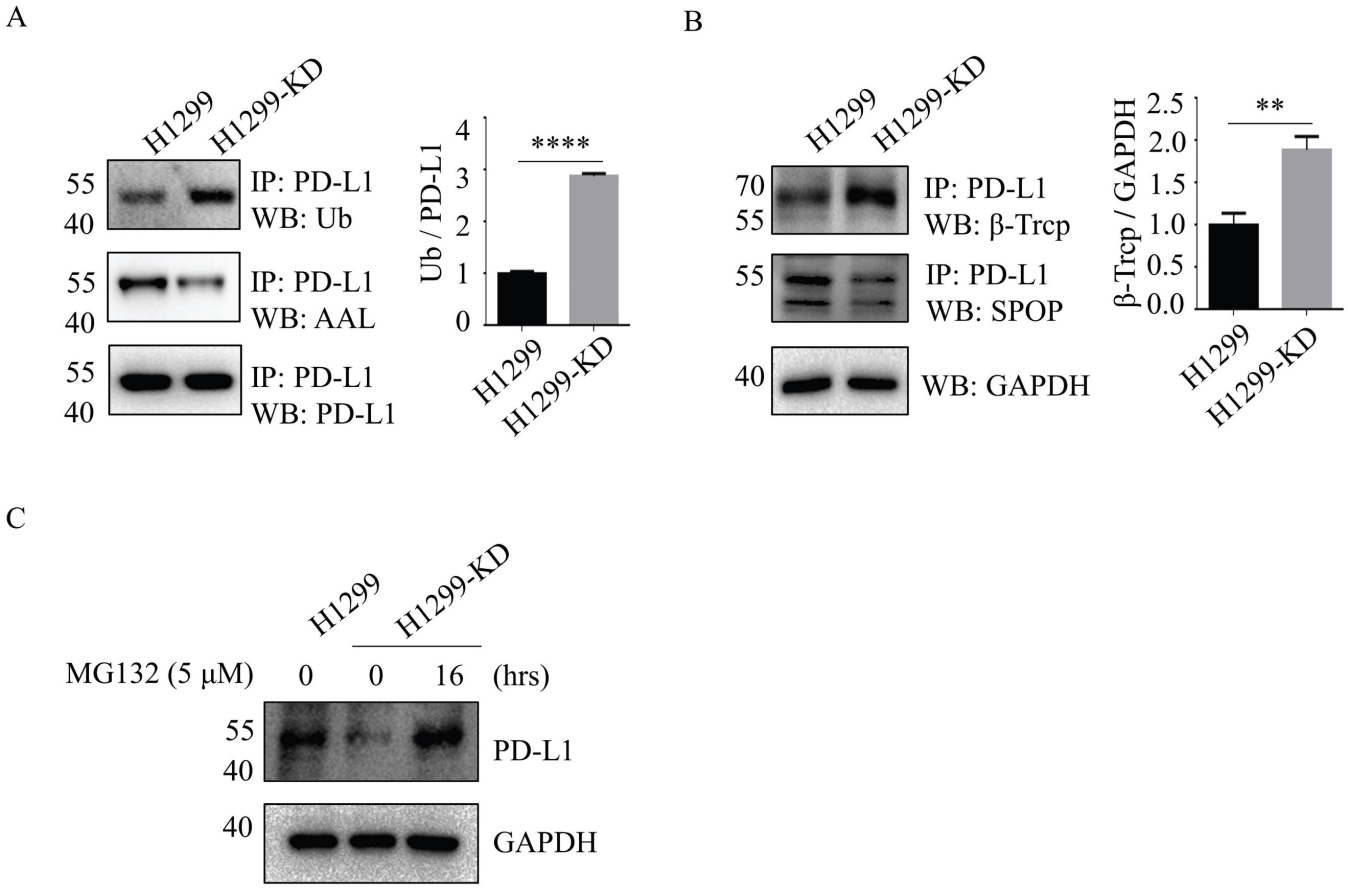
Loss of SLC35C1 promotes PD-L1 ubiquitination and its degradation. (A) Fucosylation and ubiquitination of PD-L1 in H1299 cells. Cell lysates (500 μg) were immunoprecipitated with anti-PD-L1 Ab. Immunoprecipitates were probed with anti-Ub Ab (1:200), ALL (1:12,000) and anti-PD-L1 Ab (1:1000). (B) Expression of PD-L1 E3 ligase. Cell lysates (500 μg) were immunoprecipitated with an anti-PD-L1 Ab. Protein samples were run on 10% SDS-PAGE and stained with anti-SPOP Ab (1:1,000) and anti-β-TrCP Ab (1:1,000). GAPDH was used as a loading control. (C) PD-L1 expression after MG132 treatment of H1299 cells. H1299-KD cells were treated with MG132 (5 μM) for 16 hrs. For each sample, 10 μg cell lysate was run on a 10% SDS-PAGE gel and stained with anti-PD-L1 Ab (1:1,000). GAPDH was used as a loading control. Data are shown as mean ± SD of triplicates and are representative of three independent experiments.

The ubiquitin (Ub)-proteasome system is a major cellular protein degradation machinery in eukaryotes and eliminates denatured or misfolded proteins 17. To investigate the mechanism of SLC35C1 in PD-L1 expression, we next evaluated the patterns of PD-L1 ubiquitination in H1299 and H1299-KD cells. The ubiquitination levels of PD-L1 were increased in H1299-KD cells, and corresponded to a remarkable reduction in fucosylation of PD-L1 (Fig. 3A). Previous studies demonstrated that PD-L1 is regulated by the ubiquitin/proteasome system through E3 ubiquitin ligases, such as cullin-3-SPOP (speckle-type POZ protein) and β-TrCP. Inhibition of cyclin-dependent kinases CDK4 and CDK6 increases PD-L1 protein levels by impeding cyclin D-CDK4-mediated phosphorylation of SPOP, whose binding motif is located at the 283-290 region of PD-L1 18. β-TrCP is related to phosphorylation-dependent proteasome degradation of PD-L1 14. Thus, we detected the expressions of SPOP and β-TrCP in cells. Immunoprecipitation assays with anti-PD-L1 Ab, showed knockdown of SLC35C1 markedly enhanced E3 ligase β-TrCP expression, rather than SPOP (Fig. 3B), and increased PD-1 ubiquitination in H1299-KD cells. To investigate the involvement of the 26S proteasome machinery in PD-L1 degradation, we subsequently treated H1299-KD cells with the proteasome inhibitor MG132. As anticipated, the degradation of PD-L1 was suppressed by treatment with 5 μM MG132 (Fig. 3C). These results show that loss of SLC35C1 promotes PD-L1 ubiquitination and subsequent degradation of PD-L1 by the 26S proteasome machinery.

### Loss of SLC35C1 increases β-TrCP expression by inhibiting the EGFR/ERK pathway

**Figure 4:**
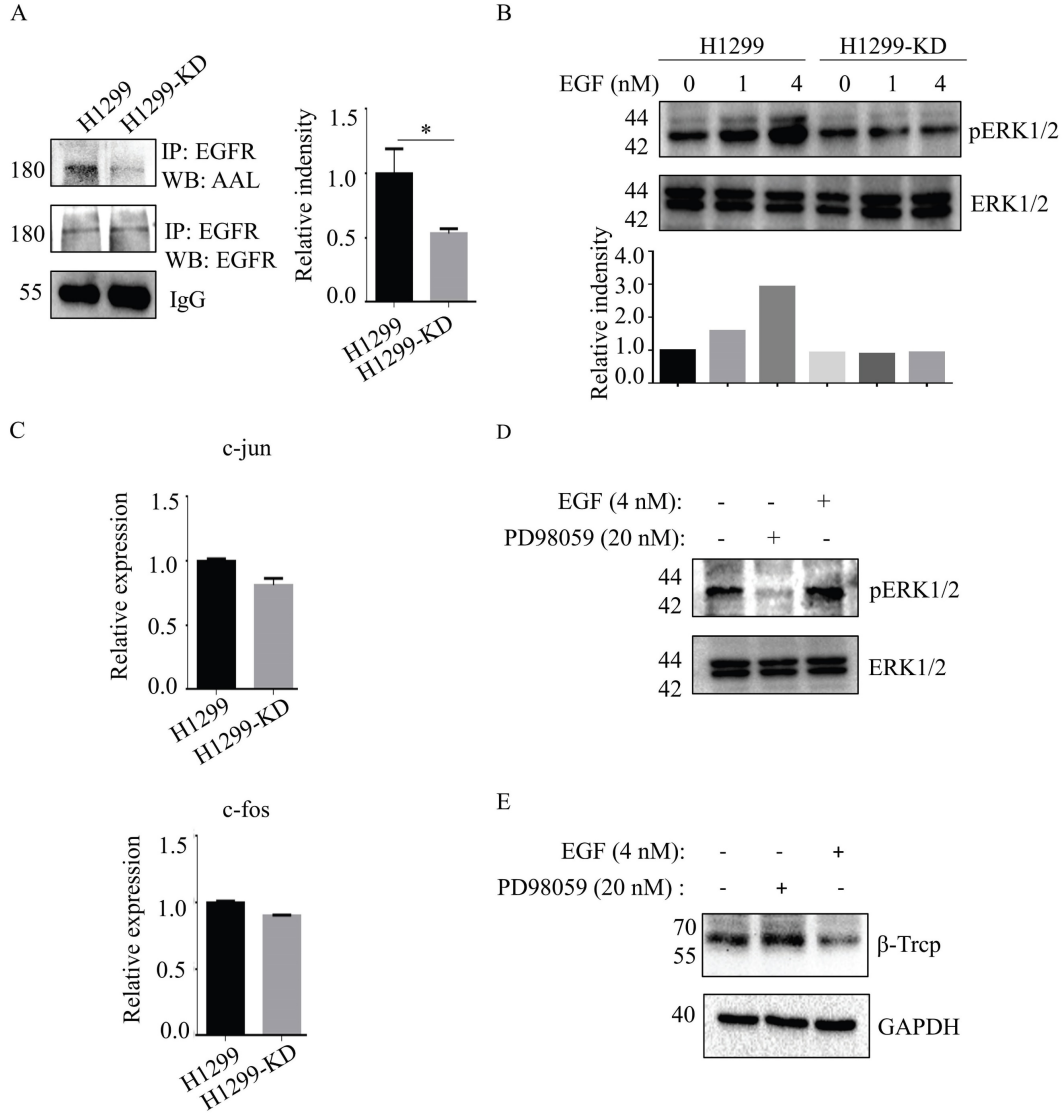
Loss of SLC35C1 suppresses activation of the EGFR/ERK pathway. (A) Low fucosylation of EGFR in H1299-KD cells. Cell lysates were immunoprecipitated with an anti-EGFR Ab. Protein samples were run on 10% SDS-PAGE. The immunoprecipitates were probed with the anti-EGFR Ab (1:1,000) and AAL (1:10,000). (B) Low phosphorylation of ERK1/2 in H1299-KD cells. Cell lysates (10 μg) were run on a 10% SDS-PAGE gel and stained with anti-ERK Ab (1:1000) and anti-pERK Ab (1:1000) after stimulation with 1 nM and 4 nM EGF for 5 min. (C) Real-time PCR. Total RNA from H1299 and H1299-KD cells was extracted with RNAiso Plus reagent. The expressions of c-fos and c-jun mRNA were detected by real-time PCR. (D) ERK phosphorylation was suppressed by cell treatment with PD98059 (20 nM). H1299 cells were treated with EGF (4 nM) and PD98059 (20 nM). Cell lysates (10 μg) were run on a 10% SDS-PAGE gel and stained with anti-ERK Ab (1:1,000) or anti-pERK Ab (1:1,000). GAPDH was used as a loading control. (E) Expression of β-TrCP was increased by cell treatment with PD98059 (20 nM). H1299 cells were stimulated with EGF (4 nM) and PD98059 (20 nM). Cell lysates (10 μg) were run on a 10% SDS-PAGE gel and stained with anti-β-TrCP Ab (1:1,000). GAPDH was used as a loading control. Data are shown as mean ± SD of triplicates and are representative of three independent experiments.

Epidermal growth factor receptor (EGFR)-mediated cellular responses to EGF regulate biological functions, including cell growth, differentiation and migration 19. EGFR contains 12 highly fucosylated N-glycans 20. Immunoprecipitation of anti-EGFR Ab showed that EGFR fucosylation was reduced in H1299-KD cells in the absence of any significant difference of EGFR expression between H1299 and H1299-KD cells (Fig. 4A). Therefore, we investigated the issue of whether a difference exists, between H1299 and H1299-KD cells, in EGF-induced EGFR activation. In contrast to H1299 cells, ERK phosphorylation was markedly attenuated in H1299-KD cells when examined at 5 min after EGF stimulation (Fig. 4B). C-fos and c-jun are well-known targets of ERK activation and play a variety of roles in many cell functions, such as cell growth and differentiation 21-23. However, SLC35C1 ablation did not change the mRNA expression of c-fos and c-jun (Fig. 4C). It has been reported that ERK regulates β-TrCP expression 24. To further confirm the function of the EGFR/ERK axis in β-TrCP expression, we treated H1299 cells with the ERK inhibitor PD98059 prior to stimulation with 4 nM of EGF. As expected, the phosphorylation of ERK was inhibited by treatment with 20 nM PD98059 (Fig. 4D), and the expression of β-TrCP was up-regulated by treatment with PD98059 (Fig. 4E), indicating that β-TrCP expression is negatively associated with ERK phosphorylation.

### Loss of SLC35C1 inhibits cell proliferation and migration by reducing PD-L1 expression

**Figure 5:**
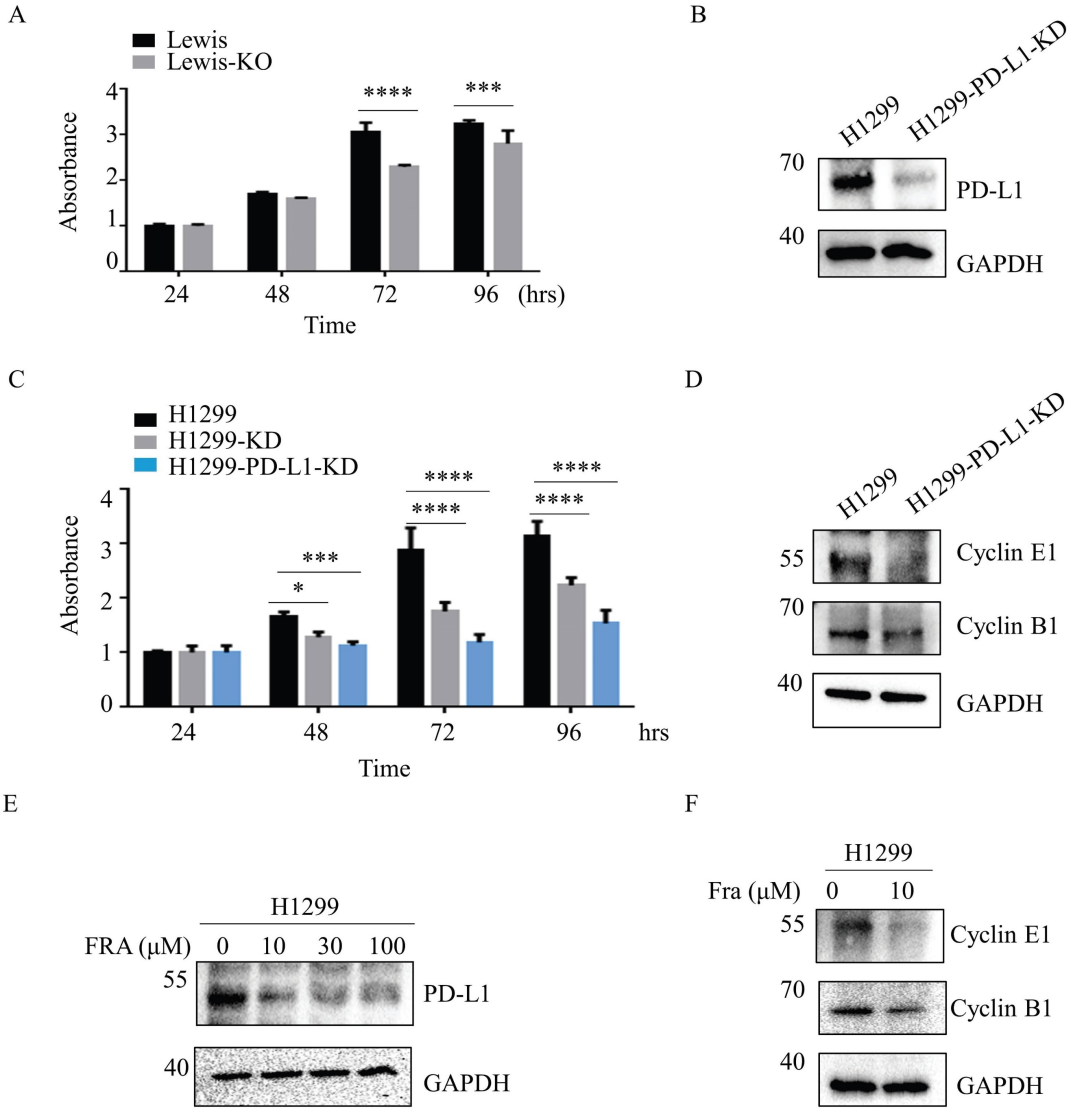
Loss of SLC35C1 inhibits the cell proliferation by reducing PD-L1 expression. (A) MTT assay. Cell proliferation of Lewis-KD cells was measured, using MTT, at 24, 48, 72 and 96 hrs. *** p<0.001, **** p<0.0001. (B) Establishment of PD-L1 knockdown H1299-PD-L1-KD cells. Cell lysates (10 μg) were run on a 10% SDS-PAGE gel and stained with anti-PD-L1-Ab (1:1000). GAPDH was used as a loading control. ** p<0.01. (C) Cell proliferation was measured using MTT in H1299, H1299-KD and H1299-PD-L1-KD cells. **** p<0.0001. (D) Expressions of cyclin B1 and cyclin E1 in H1299-PD-L1-KD cells. Cell lysates (10 μg) were run on a 10% SDS-PAGE gel and stained with anti-cyclin B1 Ab (1:1,000) and anti-cyclin E1 Ab (1:1,000). (E) H1299 cells were treated with 0, 10, 30, 100 µM FRA for 12 hrs, after which whole-cell lysates were analyzed using western blotting with anti-PD-L1 Ab (1:1000). (F) Expressions of cyclin B1 and cyclin E1 in H1299 cells following treatment with FRA. Cells were treated with 0 and 10 μM FRA, and stained with anti-cyclin B1 Ab (1:1,000) and anti-cyclin E1 Ab (1:1,000). GAPDH was used as a loading control. Data are shown as mean ± SD and are representative of three independent experiments.

Aberrant fucosylation is associated with cancer progression and metastasis 25,26. We therefore compared the effect of SLC35C1 on cell proliferation in Lewis and Lewis-KO cells. Compared to the Lewis lung adenocarcinoma cells, proliferation was suppressed in Lewis-KO cells at 72 hrs and 96 hrs (Fig. 5A). Moreover, loss of SLC35C1 also decreased cell viability of H1299 cells (Fig. 5C), suggesting that expression of SLC35C1 is required for lung cancer cell proliferation. To further investigate the role of PD-L1 in the proliferation of lung adenocarcinoma cells, we established PD-L1 knockdown (H1299-PD-L1-KD) cells (Fig. 5B). MTT assay showed that knockdown of PD-L1 markedly suppressed cell proliferation (Fig. 5C). Moreover, the expression of cyclin E and cyclin B was downregulated in H1299-PD-L1-KD cells (Fig. 5D). Fraxinellone (FRA) is a natural product that has been shown to inhibit the expression of PD-L1 in a dose-dependent manner 27. As a PD-L1 inhibitor, FRA was able to suppress the expression of PD-L1 (Fig. 5E) and also decreased the expression of both cyclin E and cyclin B (Fig. 5F), indicating that PD-L1 affects cell proliferation by suppressing the expression of cyclin E and cyclin B.

**Figure 6:**
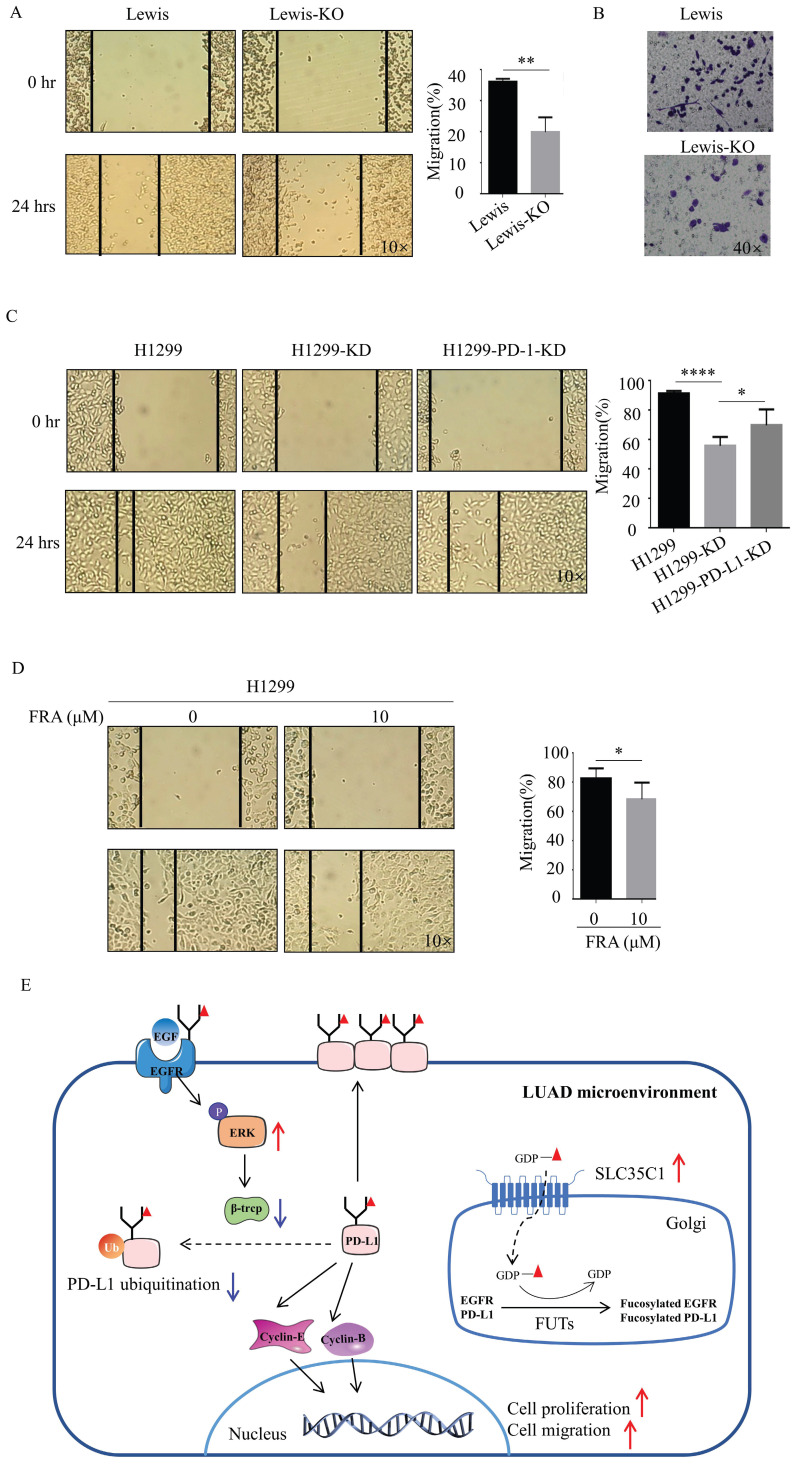
Loss of SLC35C1 suppresses cell migration by reducing PD-L1 expression. (A) Wound healing assays were performed with Lewis and Lewis-KO cells. ** p<0.01. (B) Transwell assay with Lewis and Lewis-KO cells. Cell migration assays were performed using 24-well plates with 8 μm pore size polycarbonate membrane inserts. After the cells migrated to the lower surface, the membrane was stained using crystal violet. The results were calculated by counting the stained cells under an inverted microscope. (C) Wound healing assays were performed with H1299, H1299-KD and H1299-PD-L1-KD cells, as described in Methods and Materials. * p<0.05, **** p<0.0001. (D) Wound healing assays were performed on H1299 cells treated with 0 and 10 μM FRA. * p<0.05. (E) In the LUAD microenvironment, we propose the elevated expression of the SLC35C1 gene. This overexpression of SLC35C1 contributes to the increase in PD-L1 expression via an EGFR/ERK/β-TrCP pathway to enhance cell proliferation and migration, which is a feature of LUAD tumorigenesis.

Next, we also investigated the potential effect of SLC35C1 on cell migration. Wound-healing assays showed a slow rate of wound closure in the Lewis-KO cells compared to Lewis lung adenocarcinoma cells (Fig. 6A). We also found a lower rate of wound closure in H1299-KD cells compared to H1299 cells (Fig. 6C), and knockdown of PD-L1 markedly decreased the level of cell migration (Fig. 6C). The ability to migrate was also measured using transwell assays. Loss of SLC35C1 decreased the migration of Lewis lung adenocarcinoma cells (Fig. 6B). FRA (10 μM) was able to suppress the migration of H1299 cells (Fig. 6D). These results suggest that the SLC35C1 regulates the PD-L1 expression, and cell proliferation and cell migration.

## Discussion

Hyper-fucosylation correlates with the progression of tumorigenesis, as exemplified by the high level of fucosylated-AFP in hepatocellular carcinoma 28 and core fucosylated PD-1 in lung cancer 29. In addition, the chromosomal copy number of the PD-L1 gene increases in a subset of small cell lung cancer patients and results in the massive expression of PD-L1 30. In the present study, we first found that both fucosylation and PD-L1 are upregulated in LUAD patients, and loss of SLC35C1 suppresses PD-L1 expression via the EGFR/ERK/β-TrCP pathway, which in turn decreases cell proliferation and migration (Fig. 6E).

Expression of PD-L1 in cancer cells is regulated by multiple signaling pathways, including transcription factors, such as STAT3, HIF1α and MYC. During tumor evolution, high MYC expression results in increased expression of PD-L1 by binding to the promoters of the PD-L1 gene 31. Transcription factor STAT3 binding to the PD-L1 gene promoter is also required for PD-L1 gene expression 32. However, the regulation of PD-L1 stability by ubiquitination has not yet been completely understood. In the present study, we first found that SLC35C1 ablation suppresses PD-L1 expression. Several mechanisms may contribute to the regulation of PD-L1 expression by SLC35C1. First, SLC35C1 affects PD-L1 degradation through the ubiquitin-proteasome system. Ubiquitination is a process that targets damaged proteins for degradation and modulates the localization, function and interactions of target proteins.

It has been reported that β-TrCP can influence the degradation of PD-L1 via directly ubiquitinating PD-L1 33. β-TrCP has been found to catalyze PD-L1 ubiquitination in the presence of GSK3β, which mediates phosphorylation and often facilitates ubiquitin E3 ligase recognition 14. We show SLC35C1 ablation increases the expression of β-TrCP, promotes PD-L1 ubiquitination, and reduces PD-L1 expression (Fig. 3A). Second, SLC35C1 regulates the fucosylation of PD-L1, and influences its ubiquitination. It is well known that post-translational modifications, such as N-linked glycosylation and ubiquitination of proteins, could influence protein stability and function 34. Since PD-L1 is primarily fucosylated at N^35^, N^192^, N^200^, and N^219^ 14, and also possesses ubiquitination sites 35, we speculated that fucosylation affects the ubiquitination of PD-L1. Non-glycosylated PD-L1 undergoes fast protein degradation, and among the four N-glycosylation sites, glycosylation at N^192^, N^200^ and N^219^ can contribute to PD-L1 protein stability 14. A recent study reported that deubiquitinases or associated E3 ligase inhibitors, such as hepatocyte odd protein shuttling (HOPS), can protect PD-L1 from degradation via promoting the glycosylation of PD-L1 for improving PD-L1 stability 36. Our results suggest that fucosylation ablation in the tumor microenvironment may reduce the expression of PD-L1 at the post-translational level. Moreover, PD-L1 is a trans-membrane protein that can combine with PD-1. Glycosylation of PD-1 and PD-L1 is essential for mediating their interaction 37. It has been reported that loss of core fucosylation can promote PD-1 ubiquitination and its degradation in the proteasome 29. Those studies and our current report together suggest that fucosylation, especially by SLC35C1, affects PD-L1 expression as well as function.

EGFR is an oncogenic gene and its high EGFR expression is associated with lung cancer. EGFR contains 12 N-glycans in the extracellular domain, and aberrant glycosylation impacts EGFR function. For example, the core fucosylation catalyzed by FUT8 regulates the signal pathway via EGFR 38. Additionally, FUT1 favors EGF-EGFR activation and is elevated in development of carcinogenesis and progression of cholangiocarcinoma 39. We show that knock out of the SLC35C1 gene attenuates EGFR/ERK signaling, and attenuated EGFR/ERK suppresses the expression of β-TrCP and reduces PD-L1 expression by promoting PD-L1 ubiquitination. It is noteworthy that ablation of SLC35C1 suppresses both cell proliferation and migration via reduced PD-L1 expression. Several studies have reported a major role of PD-1/PD-L1 in favoring cell proliferation and cell motility, including cell spreading, migration and invasion 40, 41. Moreover, PD-L1 deletion has been shown to inhibit cell proliferation, invasion and migration of glioblastoma cells, indicating that intracellular PD-L1 is necessary for tumor progression 42. Inhibition of PD-L1 expression in H1299 cells reduced cell proliferation and migration. Cyclins are involved in tumor growth in various cell types. Cyclin E is a nuclear protein that is essential for cell cycle progression and DNA replication, and PD-L1 mediates the regulation of expression of cyclin E 43. Cyclin B1 expression peaks during G2 to M transition and it is a crucial cell cycle checkpoint protein that promotes mitosis, and is overexpressed in a variety of tumors 44. In the present report, we found PD-L1 inhibition decreases the expression of both cyclin E and cyclin B. SLC35C1 silencing hinders the proliferation, migration and invasion of trophoblast cells, but the regulatory mechanisms still need to be delineated 45. Our results suggest that, in the LUAD microenvironment, anomalous fucosylation is linked to PD-L1 expression and contributes to the proliferation and migration of lung cancer cells.

PD-L1 is regarded as the primary ligand of PD-1, which is mainly expressed on different immune cells, such as T cells. Recent studies have shown that PD-1/PD-L1 can influence tumor progression as a vital immune checkpoint in the tumor microenvironment, and over-expression of PD-1 and PD-L1 contributes to tumor immune escape 46. Antibodies targeting PD-1 and PD-L1 can block PD-1/PD-L1 interaction and have been used to treat tumors clinically by reversing tumor-mediated immunosuppression 47. We first clarify the potential regulatory mechanism of how SLC35C1 regulates PD-L1 expression, cell proliferation and migration in LUAD. SLC35C1 can be considered as a potential novel target for lung cancer treatment.

## Supplementary Material

Supplementary table.
