# Supplementary material for: Ablation of the GDP-fucose transporter suppresses lung cancer cell proliferation and migration by reducing expression of PD-L1
Source: J Cancer. 2023 Oct 2;14(17):3295–308. doi: 10.7150/jca.84652 (PMC10623000; doi:10.7150/jca.84652)
Supplement: Supplementary file 1 — Supplementary table. [file jcav14p3295s1.pdf]

Supplementary files

Table S1. Correlation expression of PD-L1 in lung adenocarcinoma tissues

Table S1. Correlation expression of PD-L1 in lung adenocarcinoma tissues

Characteristic	Total (n=92)(%)	PD-L1 expression		P-value
		high	low	
Age (years)				
< 65	52 (56.52%)	27	25	0.6709
≥ 65	40 (43.48%)	22	18	
Sex				
Male	51 (55.43%)	28	23	0.7759
Female	41 (44.57%)	21	20	
Pathologic grade				
I-II	61 (66.30%)	28	33	<0.005
III	31 (33.70%)	21	10	
TNM stage				
I-II	58 (63.04%)	30	28	0.8876
III-IV	34(36.96%)	17	17	
AJCC stage				
I	36 (39.13%)	21	15	0.2022
II-IV	56 (60.87%)	27	29	
